# School-based cognitive behavioural intervention programme for addressing anxiety in 10- to 11-year-olds using short classroom activities in Japan: a quasi-experimental study

**DOI:** 10.1186/s12888-022-04326-y

**Published:** 2022-10-25

**Authors:** Yuko Urao, Michiko Yoshida, Yasunori Sato, Eiji Shimizu

**Affiliations:** 1 Research Centre for Child Mental Development, Chiba University, 1-8-1 Inohana, Chuo-ku, Chiba, 260-8670 Japan; 2 Department of Cognitive Behavioural Physiology, Chiba University Graduate School of Medicine, 1-8-1 Inohana, Chuo-ku, Chiba, 260-8670 Japan; 3 Department of Preventive Medicine and Public Health, Keio University School of Medicine, 35 Shinanomachi, Shinjuku-ku, Tokyo, 160-8582 Japan

**Keywords:** Prevention, Cognitive behavioural intervention, Anxiety, School-based

## Abstract

**Background:**

Although several school-based cognitive behavioural intervention programmes have been developed in Japan to prevent and improve children’s anxiety disorders, the substantial time required for their completion remains a problem.

**Methods:**

A brief version of the cognitive behavioural programme called ‘Journey of the Brave’, developed for Japanese children was conducted among 90 children aged 10‒11 years using 20-min short classroom activities, and its effectiveness was examined. The children were divided into two groups: the intervention (*n* = 31) and control groups (*n* = 59). The control group did not attend any programme sessions and followed regular school curriculum. We conducted 14 weekly programme sessions and assessed children at pre-intervention, post-intervention, and 2-month follow-up (6 months after the beginning). The primary and secondary outcome measures were the Spence Children’s Anxiety Scale (SCAS) to assess children’s anxiety symptoms and the Strengths and Difficulties Questionnaire (SDQ) to measure behaviour problems, respectively.

**Results:**

A statistically significant reduction in the SCAS score in the intervention group was found at 2-month follow-up compared with the control group. A significant reduction was also observed in the SDQ score.

**Conclusions:**

Our findings suggested that the ‘Journey of the Brave’ programme, which requires only 5 h of short classroom activities, demonstrates promising results compared with previous programmes. A larger randomised control trial would be desirable.

**Trial registration:**

UMIN, UMIN000009021, Registered 10 March 2012.

## Background

Anxiety disorder is one of the most prevalent psychological disorders experienced in childhood and adolescence [1–3]. Some studies have demonstrated that the 6 to 12-month prevalence of anxiety disorders among children and adolescents is approximately 10% [4–6]. Children with anxiety are prone to maladaptation at school. For example, they experience panic attacks, fatigue, or sleep disturbance from anxiety, which may lead to underachievement [7]. Additionally, those with low self-esteem may avoid social relations and have negative peer interactions [8], resulting in school absenteeism [9].

Although the current record of school absenteeism in Japan, by all available measures, has not reduced over time, in 2014, Japan’s Ministry of Education [10] reported that the primary reason for absenteeism in elementary schools was ‘emotional confusion (e.g. anxiety)’. Additionally, Ishikawa et al. [11] associated school absenteeism with anxiety and depression. Children with developmental problems including attention deficit hyperactivity disorder and Autism Spectrum Disorder often express symptoms of anxiety [12]. If left untreated, anxiety in childhood can lead to an increased risk of other psychological disorders, the impairment of development, and disturbances in career domains [13–15]. Therefore, prevention and early intervention is important to prevent problems in adulthood [16–18].

Schools offer an optimal setting for interventions to prevent and reduce anxiety among children [19–21]. Primary interventions can be defined as either universal, selected, or indicated [22]. The school setting is an ideal environment for universal intervention targeting the entire student population [23, 24]. Schools have several advantages, such as enabling easier access to programme sessions, reducing stigmatisation, and enhancing peer support [25–27].

Cognitive behavioural therapy (CBT) is a recommended treatment choice for anxiety disorders among children [28–30]. CBT-based anxiety prevention programmes have been developed in other countries, and their effects have been demonstrated in meta-analysis studies in universal interventions at schools [31, 32]. Among them, one of the most widely recognised school-based CBT programmes is the ‘FRIENDS’ programme which aims to decrease anxiety and to promote resilience of children [33]. FRIENDS has gained recognition as an effective programme for the reduction and prevention of anxiety and depressive symptoms by the World Health Organisation [34] with several significant anxiety reduction results. For example, Neil and Christensen [35] reported that the effect size of the FRIENDS programme targeting anxiety at follow-up was 0.33–0.41 and 0.62 in both Barrett and Turner’s [36] and Lowry-Webster et al.’s [37] studies.

Although FRIENDS was developed in Australia and its implementations and effectiveness verification studies have been conducted worldwide, predominantly in Western countries [36–39], it is rarely tried in Japan. It is difficult to directly import programmes developed in Western countries to Japanese schools owing to socio-cultural differences [40]. Therefore, a Japan-original CBT-based programme ‘Journey of the Brave’ for Japanese children was independently developed, and an initiative to examine the effects [40] began in 2014. The initial trial was conducted outside the school setting as a preliminary study, and the results demonstrated that the programme was effective based on parents’ evaluations. Thereafter, intervention studies in elementary schools have been conducted showing significant programme effectiveness [41].

### Current study

Despite the availability of an effective CBT-based intervention for childhood anxiety that is applicable to the school setting, there are several issues to its implementation in schools in Japan.

The first issue is the session length of the programme [42, 43]. Studies report that students’ attention declines in the first 10‒15 min of a lecture [44]. The session length must be short enough to maintain children’s concentration. Each session of the universal approach programme usually lasts from 60–90 min in other countries [35], and it is estimated that the total time required to complete the programme is 10–15 h. The initial trial of the ‘Journey of the Brave’ programme [40] comprised ten 45-min sessions, taking 7.5 h to complete. As schools have other academic requirements and school events, pragmatic challenges such as space and time availability in schools must be addressed when providing mental health programmes [42, 45]. Using the time frame of short classroom activities in implementing the ‘Journey of the Brave’ programme is advisable to address the issue of programme length.

The second issue concerns the time of the day when the program is implemented. A study evaluated the effects of the after-school CBT programme on the anxiety of children with high anxiety symptoms [46]. According to a child self-report, the study results indicated no significant effects on symptoms of anxiety among children who received the CBT programme. The result of their study was partially explained by high absenteeism owing to delivering the programme after school. A report on adult stress has indicated that the mind does not experience equal levels of stress and conflict in the morning and the evening [47, 48]. Thus, it is beneficial to implement the programme as part of short class activities in the morning in a school schedule.

The third issue is regarding the age at which the program is implemented. Most elementary schools in Japan have class shuffles when children are promoted from the 4th (9–10 years old) to the 5th (10–11 years old) grade. This environmental change poses a negative mental health effect on children, causing increased anxiety [49, 50]. Thus, it is necessary to build new friendships, and most children might be anxious during this period. Additionally, Grade 5 (10- to 11-year-olds) children have a new responsibility as school leaders and meet increased opportunities for social communication, forcing them to face higher pressure and anxiety after the class re-shuffle. Thus, the programme to manage anxiety, which comprises the necessary contents for 10- to 11-year-old children, may match their will to learn and deepen their understanding of contents.

Considering the aforementioned aspects, this study aimed to implement and examine the effectiveness of the CBT-based programme intervention ‘Journey of the Brave’ using short classroom activities among 10- to 11-year-old children.

## Method

### Procedure

#### Participants and informed consent

**Fig. 1:**
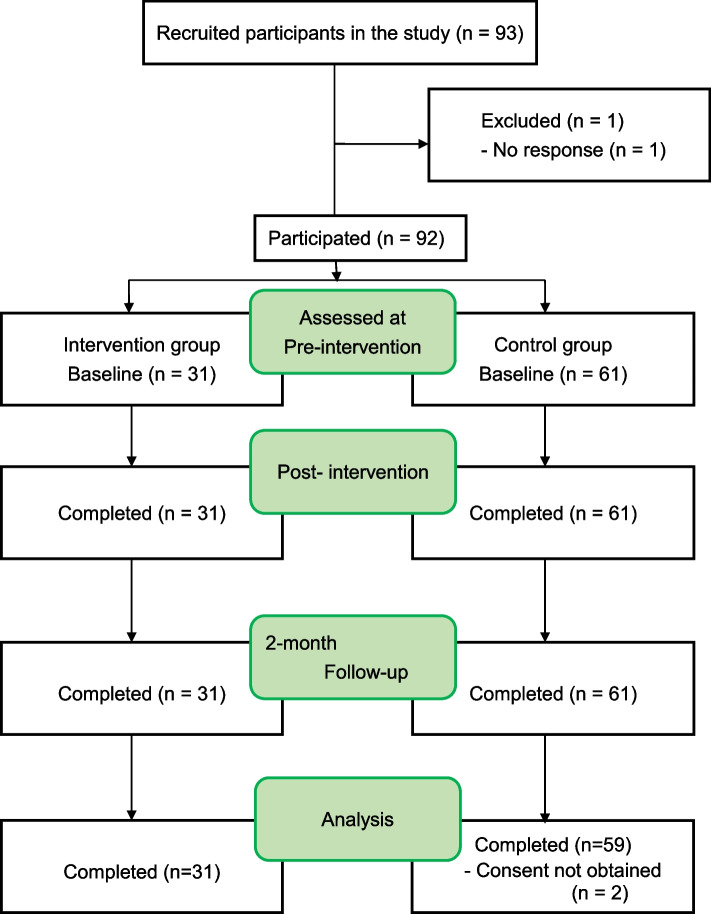
Flow-chart shows the number of children recruited, dropout in the evaluation at each point, and sample count of MMRM. Abbreviations: MMRM, mixed-effect model for repeated measures

Our team member, who was working in a school in the vicinity of Tokyo as the nurse teacher, explained the study protocol and the schoolmaster granted permission for data collection. As it was compulsory for children to receive class sessions in each class, it was impossible to randomly assign the intervention and control groups. Thus, the approval of the schoolmaster was received with the condition that non-randomised design was applied assigning one of three 5^th^ grade classes as the intervention while the remaining two as the control group. The cross-over design was applied whereas the control group also took programme sessions later. Programme explanations with consent forms were delivered through the children to their parents, who provided consent on behalf of their children. The completed consent forms were returned to the school. A total of 93 consent forms were distributed and 92 returned (57 male participants and 35 female participants) with parent’s consent. One child did not respond because of a psychological block about coming to school (Fig. 1).

#### Intervention group

A class consisting of 31 (19 boys, 12 girls) children, with an average age of 10.0 (SD = 0) received 14 weekly sessions of ‘Journey of the Brave’ once a week. Each child was provided a workbook that they used throughout the programme.

#### Control group

Prior to programme commencement for the intervention group, the parents of children from the control group in two classes consisting of 61 children (36 boys, 23 girls) with the average age of 10.1 (SD = 0.3) were explained that the control group will attend the sessions later. Two children were unable to obtain signed consent forms from their parents. During the programme, children in the control group received the regular school curriculum (e.g. reading, calculation exercises) facilitated by the classroom teacher.

#### Programme facilitators and training

The ‘Journey of the Brave’ programme was led by two facilitators. The main programme facilitator was the nurse teacher of the school and was majoring in Cognitive Behaviour Physiology as a graduate student at the time of the study. We designated the teacher in charge of this class as the sub-facilitator of the programme. Prior to conducting the programme, the facilitators were provided t hours’ training once every 2 weeks for 3 months by the first author who developed the original programme. During the implementation phase, the facilitators contacted the first author once a week to discuss problems and/or questions that arose during the intervention sessions, and received supervision and support.

#### Description of journey of the brave programme

The ‘Journey of the Brave’ programme was developed for 9- to 12-year-old children, focussing on feelings of anxiety and the key behaviour changes to manage these feelings [40]. It comprises multiple 10 45-min-long sessions. The programme contents are based on the protocol of CBT for anxiety treatment. It includes contents such as psychological education, relaxation, case formulation of anxiety, exposure, cognitive restructuring, and assertiveness skills when faced with social anxiety. The largest difference between this programme and existing CBT-based ones is that the content was developed based on the socio-cultural background of Japan. For example, while many of the FRIENDS contents include group activities, the ‘Journey of the Brave’ programme is constructed with an individual study format because several children in Japanese schools experience social anxiety, and group activities increase their anxiety. Additionally, the illustrations and characters in the programme workbook are familiar to Japanese children. Moreover, each session is designed to take less than 45 min and a teacher-friendly manual is prepared.

### Programme implementation using short classroom activities time

In Japan, there are short classroom activities in the morning as part of regular curriculum in most elementary schools. However, the way this time is used differs in every school, and in many cases, the time allocated for this is 20 min (8:20–8:40 for example). The activities include both active physical movement and passive learning such as reading and calculation. Individual content is decided by each school as long as it fits the purpose of children’s overall health promotion. Therefore, mental health programmes such as the one introduced here is quite appropriate.

Therefore, to implement the ‘Journey of the Brave’ in the 20-min class morning activities time slot in school in Japan, we divided each session into two. Additionally, we decided to not include the session on assertiveness skills, because the same theme had already been handled in the existing school curriculum. The cognitive restructuring session was allotted the entire 45 min to ensure children’s comprehension. Thus, the number of sessions of this programme increased to 14; however, the total implementation time decreased to approximately 5 h (Table 1).

**Table 1:** Outline of the anxiety prevention program in this study

Session	Content	Study objective *Exercise focus
1–4	Understanding feelings of anxiety	Understanding that anxiety is an important feeling that protects one from danger and that it is not necessary to totally eliminate anxiety ^*^Clarify anxiety object and set a target
5	Body reactions and relaxation	Learning that anxiety and tension of both body and mind can be reduced by relaxation ^*^Practice and acquire techniques of breathing and muscle relaxation
6–7	Anxiety level stages and stair step exposure	Learning that it is important to gradually expose oneself to anxiety rather than to avoid it ^*^Develop anxiety hierarchy table ^*^Climb anxiety ladder step by step (up to Session 14)
8–9	Anxiety cognition model	Learning that cognition, behaviour, and feelings are closely connected to each other and that the level of anxiety changes with cognition ^*^Develop a triangle of cognition, behaviour, and feelings
10–11	Rumination of the maladaptive cognition	Learning that anxiety will inflate by the rumination of the maladaptive cognition and lead to a vicious circle ^*^Realize maladaptive cognition
12 (45 min)	Cognitive restructuring when anxious	Learning that anxiety can be reduced by reviewing and restructuring cognitions when anxious ^*^Restructure cognition at anxious moments
13	Review	Reviewing each session’s content with all participants ^*^Reviewing sessions 1 to 8
14	Summary	Confirming how anxiety level and self- confidence have changed by participating in the ‘Journey of the Brave’ programme ^*^Graduation ceremony

### Measurements

The participating children completed a set of sequential questionnaires during the session at three different time points: pre-intervention, post-intervention, and 2-month follow-up. Except for the evaluation form, they completed the self-report measures during their regular class time. The parents of the intervention group children received the evaluation form at home through their children, and they returned the forms to the school after completion.

### Quantitative data to verify effectiveness

#### Spence children’s anxiety scale (SCAS)

The primary outcome measure was the anxiety symptoms reported by children, measured using the Spence Children’s Anxiety Scale [51], one of the most valid measurements for assessing child anxiety meeting the diagnostic standard. The questions are applicable to 8- to 15-year-old children, and good reliability and validity coefficients of the SCAS Japanese version have been reported [52]. The SCAS includes 38 items regarding children’s anxiety symptoms divided into six subcategories: separation anxiety, social phobia, panic disorder/agoraphobia, generalised anxiety disorder, physical injury fears, and obsessive–compulsive disorder. SCAS scores range between 0 (never) and 3 (always), and the maximum possible score is 114.

#### Strengths and difficulties questionnaire (SDQ)

The secondary outcome measure was behaviour problems, measured using the self-report version of the Goodman SDQ [53]. The questions were applicable to 4- to 16-year-old children. Reliability and validity coefficients of the Japanese versions of the SDQ have been reported [54]. The SDQ includes 25 items, with each item scored 0 (not true), 1 (somewhat true), or 2 (certainly true), according to the perceived severity of the symptom. The items are divided into five subcategories: emotional symptoms, behaviour problems, hyperactivity/inattention, peer relationship problems, and pro-social behaviour. A total difficulties score (TDS) is computed by summing the scores of the first four subcategories, and the maximum possible score is 40.

### Qualitative data to assess acceptability and feasibility

#### Programme evaluation form for children

This form was used to measure children’s acceptance and satisfaction with the programme. Participants were asked to rate the extent to which they could understand the components of this programme and how helpful the skills they learned were in their daily life. This form utilised a four-point Likert-scale, and the respondents were asked to rate each item according to their own experience; the response options were ‘yes’, ‘a little’, ‘not really’, and ‘no.’ A numerical equivalent was assigned to each answer, and percentages were analysed. Additionally, children were asked to write free comments about the programme. Furthermore, the programme sub-facilitator asked the children to provide free comments on the quality and feasibility of the programme after the intervention.

#### Programme evaluation form for parents

This form was used to assess the parents’ evaluation of the programme and its perceived effects on their children. Parents were asked how helpful they thought the programme was for their children and how this program met their expectations. This form used a four-point Likert-scale, with the response options and analysis methods being the same as that in the evaluation form for children. Free comments were also obtained about the programme.

### Statistical analysis

For the baseline variables, summary statistics were constructed using frequencies and proportions for categorical data, and means and standard deviations (SDs) for continuous variables. The participant characteristics were compared using a chi-square test for categorical outcomes and a t-test or the Wilcoxon rank sum test for continuous variables, as appropriate. Additionally, t-tests were conducted on both SCAS and SDQ at baseline to confirm the difference between groups.

To confirm the SCAS and the SDQ score changes at 2-month follow-up from baseline, primary analysis was performed using the mixed-effects model for repeated measures (MMRM) with intervention group, time (week), and interactions between treatment group and time (week) as fixed effects; an unstructured covariate was used to model the covariance of within-subject variability. MMRM analysis used all the available data and assumed that any missing observations were missing at random. Under the ignorable missing data framework, MMRM analysis is a robust approach for estimating the true treatment difference and controlling Type I error rates [55, 56]. However, when data are not missing at random, these inferential techniques that are valid for missing-at-random data are typically invalid [57, 58].

To compare the efficacy and effectiveness of the intervention programme, SCAS and SDQ effect size (ES) estimates were calculated using Cohen’s d [59]. Cohen’s d values were calculated as the difference between the intervention and the control groups’ means divided by the pooled standard deviation. According to Cohen [59], an effect size of 0.2 is considered small, 0.5 is considered medium, and 0.8 is considered large.

All statistical tests were two-tailed, and a *p*-value of 0.05 was employed. Other statistical analyses were performed with IBM SPSS Statistics for Windows, Version 17.0 (IBM, Armonk, New York, USA), and SAS software version 9.4 (SAS Institute, Cary, NC, USA).

## Results

A total of 90 children (31 and 59 in the intervention and control groups, respectively) completed the questionnaire, and their data were used for analyses (Fig. 1). The differences in gender and age were analysed between the intervention and control group children at pre-test. There were no significant differences (Table 2). Next, to compare the differences in baseline SCAS scores at pre-test between the intervention and control groups, t-tests were conducted. There were no significant differences in SCAS scores (Table 2). T-tests were also conducted to compare the differences in baseline SDQ scores at pre-test between the intervention and control groups. Although there was no significant difference in the SCAS score, significant differences in the SDQ scores were observed (Table 2).

**Table 2:** Participants’ demographic and baseline outcome data

	**Intervention (*n* = 31)**	**Control (*n* = 59)**	**Test**	***p*-value**
Gender Female	*n* = 12 (39%)	*n* = 23 (39%)	x^2^ = .001	0.98
Age	10.0 (0.00)	10.1 (0.30)	x^2^ = 3.38	0.07
SCAS	17.77 (14.96)	23.83 (13.96)	t [88] = 1.91	0.60
SDQ	8.71 (4.13)	10.95 (4.99)	t [88] = 2.14	0.04*

*Abbreviations*: *SCAS* Spence Children’s Anxiety Scale, *SDQ* Strengths and Difficulties Questionnaire, *SD* Standard deviation

Note. * *p* < 0.05

**Fig. 2:**
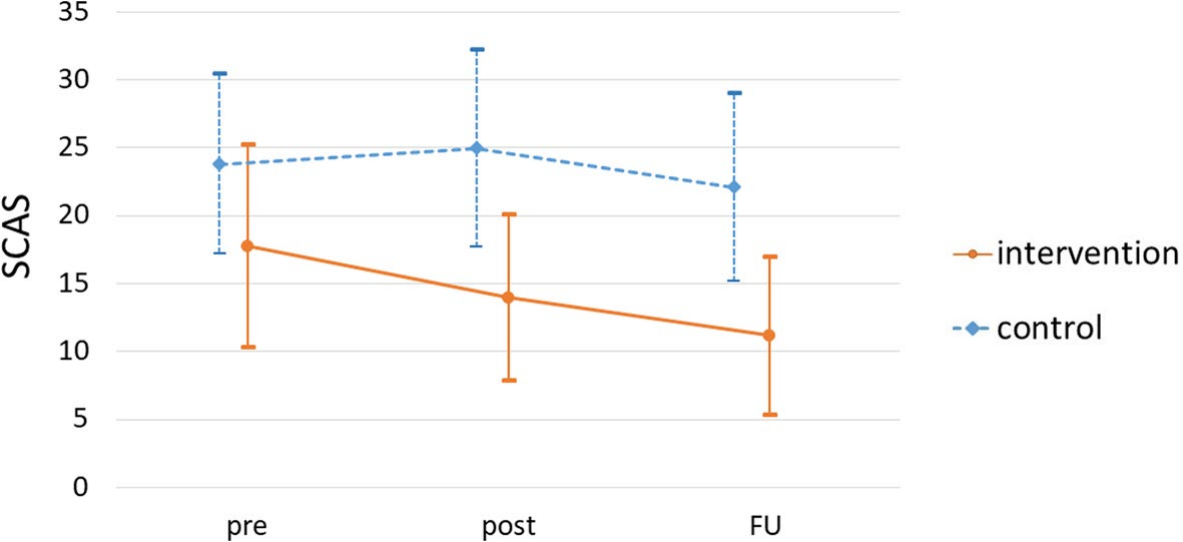
Mean total SCAS score change of each group during the study. shows the SCAS score of the intervention group and the control group for each time period. Abbreviations: SCAS, Spence Children’s Anxiety Scale

The SCAS score changes are indicated in Fig. 2 and Table 3. At 2-month follow-up, the estimated mean reductions in SCAS from baseline by MMRM were -7.17 in the intervention group (95% CI = -10.64 – -3.70) and -1.40 in the control group (95% CI = -3.91 – 1.11), and the between group difference was -5.77 (95% CI = -10.07 – -1.47, *p* = 0.009; Table 4). The effect size (Cohen’s d) estimate at 2-month follow-up was 0.46 between the intervention and control groups.

**Table 3:** SCAS and SDQ score at each time point

		**Intervention (*n* = 31)**	**Control (*n* = 59)**				
		Pre	Post	FU	Pre	Post	FU
**SCAS**	Mean (SD)	17.77 (14.96)	13.97 (12.24)	11.19 (11.63)	23.83 (13.96)	25.05 (15.25)	22.07 (14.54)
**SDQ**	Mean (SD)	8.71 (4.13)	8.32 (4.33)	7.29 (3.76)	10.95 (4.99)	11.08 (5.32)	10.19 (4.66)

*Abbreviations*: *SCAS* Spence Children’s Anxiety Scale, *SDQ* Strengths and Difficulties Questionnaire, *FU* Follow-up, *SD* Standard deviation

**Table 4:** Estimated changes at 2-month follow-up from baseline on SCAS and SDQ scores by mixed-effects models for repeated measures

	**Group**	**Estimated mean**	**Group difference**	**Between group difference (95% CI)**	***p*-value**
**SCAS**	Intervention	-7.17	-5.77	-10.07 ― -1.47	0.009**
	Control	-1.40			
**SDQ**	Intervention	-1.81	-1.60	-2.98 ― -0.21	0.024*
	Control	-0.22			

*Abbreviations*: *SCAS* Spence Children’s Anxiety Scale, *SDQ* Strengths and Difficulties Questionnaire

Note. * *p* < 0.05, ** *p* < 0.01

**Fig. 3:**
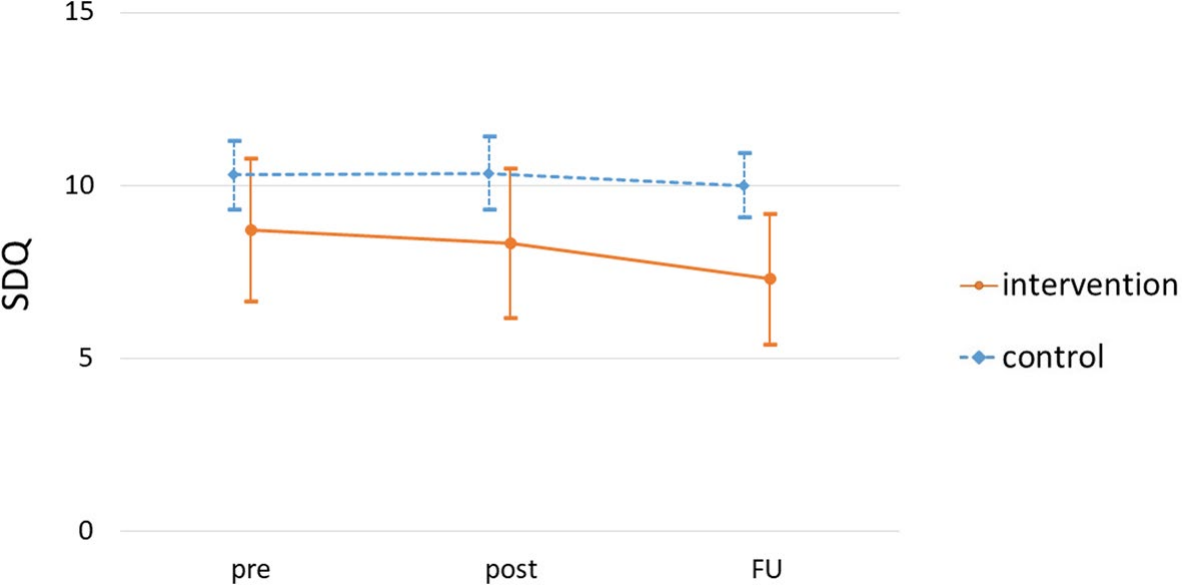
Mean total SDQ scores in each group during the study. shows the SDQ score of the intervention and control groups for each time period. Abbreviations: SDQ, Strengths and Difficulties Questionnaire

The SDQ score changes are indicated in Fig. 3 and Table 3. At 2-month follow-up, the mean reductions in the change of SDQ scores from baseline were -1.81 in the intervention group (95% CI = -2.93 – -0.70) and -0.22 in the control group (95% CI = -1.02 – 0.59), and the between- group difference was -1.60 (95% CI = -2.98 – -0.21, *p* = 0.024; Table 4). The effect size (Cohen’s d) estimate at 2-month follow-up was 0.15 between the intervention and control groups.

### Evaluation form to assess acceptability and feasibility

Evaluation forms for children were returned by all the children (*n* = 31) in the intervention group. In total, 81% of children responded that they were able to cope with anxiety by themselves, and 84% responded that the skills they learned during the programme will be helpful in daily life. Additionally, 94% of the children were satisfied with learning the programme in the school setting.

**Fig. 4:**
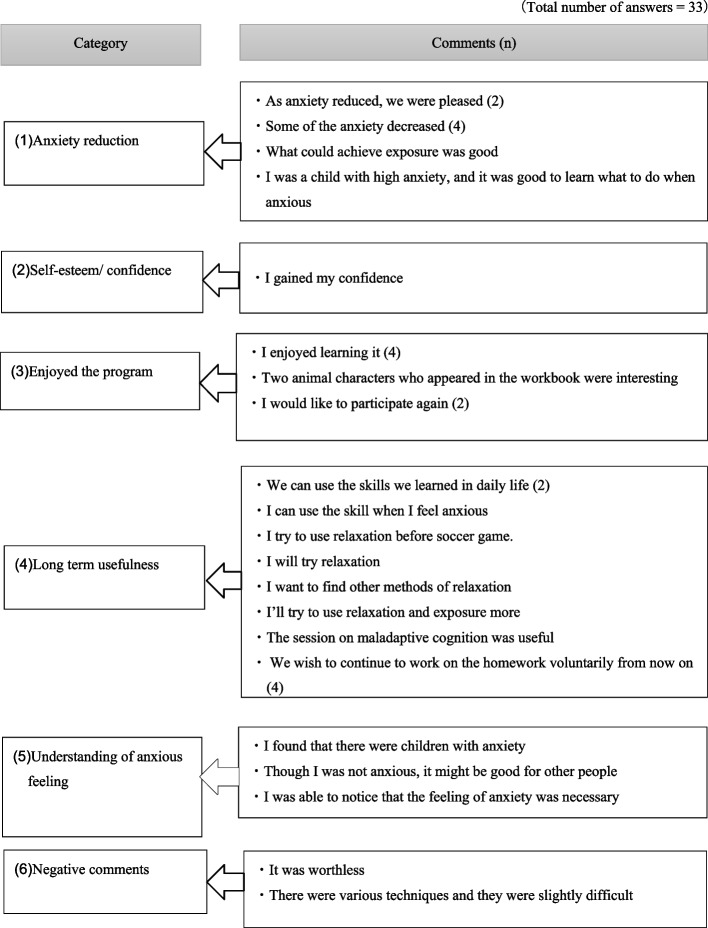
Excerpts from free comments on the ‘Journey of the Brave’ programme (intervention group children). presents some of the free comments from the intervention group children about the programme after the intervention

**Fig. 5:**
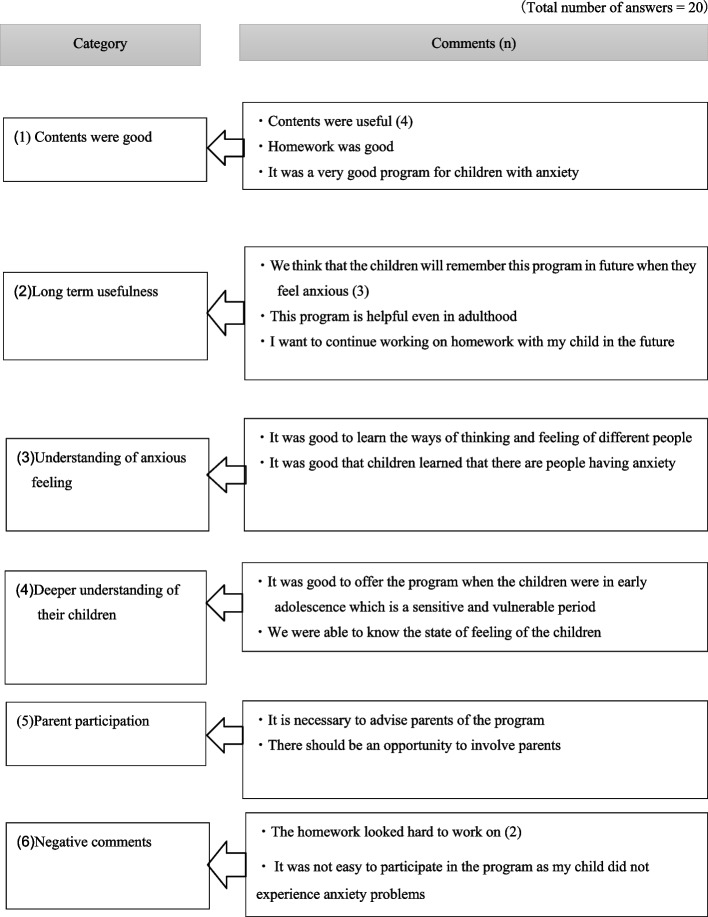
Excerpts from free comments on the ‘Journey of the Brave’ programme (intervention group parents). presents some of the free comments from the intervention group parents about the programme after the intervention

Evaluation forms for parents were returned by 29 parents in the intervention group. In total, 86% of them responded that the skills that their children learned during the programme would be helpful in their daily life, and 97% felt that the programme should be implemented in school settings. Excerpts of free comments about the programme in the intervention group are provided in Figs. 4 and 5.

Furthermore, the sub-facilitator evaluated the programme as follows:

- The timing was good because it was after the class shuffle.
- Conducting the exposure task progressively, was especially adequate for children.
- The session pertaining to maladaptive cognition was helpful in classifying the cognitive style of the children.
- I would like to receive feedback and enhance the skills learned during the programme to apply them to school life.

## Discussion

The results of our study showed a statistically significant reduction in the intervention group at 2-month follow-up in anxiety symptoms and behaviour problems compared with the usual care control group despite the programme being delivered in a short and divided time format. This result confirms the positive effect shown in the previous pilot study [40] as well as the following effectiveness verification studies [41, 60].

The original ‘Journey of the Brave’ programme took 10 weekly sessions, with each session lasting 60 min compared with that of our study with a duration of only five hours. Additionally, the qualitative data received in the evaluation form were mostly positive, and we judged that there was no problem in the acceptability and feasibility of the programme, indicating its efficiency. The result, if verified under more robust research design, may help overcome some barriers to implementing this in Japan because it can be smoothly incorporated into the school timetable and saves manpower.

However, the numerous limitations of this study should be noted. First, there was the issue of recruitment bias. As it was difficult to identify the participating school, the school where one of our research team members was working as the nursing teacher was designated as the study field; moreover, she served as the facilitator of the programme. The possibility of daily relationships between the teacher and the children may have impacted our results. Additionally, we employed a nonrandomised control design. It was impossible to evade the contamination risk between the intervention and control groups as children from the same grade year and school were selected as the control group. Conducting a study with a randomised control design is recommended to establish evidence of CBT practices. Therefore, it is necessary to conduct a study with a randomised block control design by removing moderators and mediators of anxiety in the future (e.g., characteristics of class and school events).

Second, important factors such as reliability of the facilitator and programme fidelity are not explored. The facilitator of this study had acquired the ‘Journey of the Brave’ facilitator qualification, had completed the training course of Improving Access to Psychological Therapies, and was studying CBT. She was familiar with the programme as she was one of the members of the research team receiving advice and supervision from the programme author once a week. However, we were unable to quantitively evaluate reliability and programme fidelity because objective measures were not used. In the future, using a device to monitor the facilitator who is implementing the programme is necessary.

Finally, this study does not report beyond the 2-month follow-up time point. Further research is needed to investigate longer follow-up durations (e.g. 1-year, 2-years) to determine the impact of the programme over time. It has been indicated that children are at risk for emotional and behavioural problems during the transition time when they advance from elementary school to junior high school [61]. Therefore, evaluation during this period is important to determine the long-term effects of this intervention.

As stated above, rigorousness of this study is missing on multiple grounds. Hereafter, we wish to secure greater visibility and credibility with a more robust study design to disseminate findings of superior quality.

## Conclusions

This study demonstrated that the delivery of the ‘Journey of the Brave’ programme in the form of brief classroom activities results in a significant reduction in anxiety symptoms and behavioural problems. Our findings suggest that the programme, which is completed in only 5 h, is as effective as programmes that have taken over 10 h to complete in previous studies. The significant room for improvement in the current study warrants future research with improved designs.

## Data Availability

The datasets used and/or analysed during the current study are available from the corresponding author on reasonable request.

## Abbreviations

CBT: Cognitive Behavioural Therapy
ADHD: Attention Deficit Hyperactivity Disorder
SCAS: Spence Children’s Anxiety Scale
SDQ: Strengths and Difficulties Questionnaire
MMRM: Mixed-effect model for repeated measures
